# Measures for Checking the Vomiting Excited by Anæsthetics

**Published:** 1893-04

**Authors:** 


MEASURES FOR CHECKING THE VOMITING EXCITED BY ANÆSTHETICS.—The *Union medicale* for February 28th has a short article on this subject, relating more particularly to cases in which chloroform is the anærthetic employed. The first measure mentioned for arresting chloroform vomiting is to increase the anæsthesia to the point of abolishing all reflex sensibility. This, the writer says, does not always succeed; he might have added that on other grounds it was not to be commended. Another plan, proposed by Joos, of Winterthur, but said by the writer to have been borrowed from Leloir, of Lille, who used it in cases of intractable hiccough, is that of compression of one or both of the phrenic nerves by means of the thumb placed immediately above the sternal end of the clavicle. The compression should be kept up for several minutes after the vomiting has ceased. If it does not succeed, recourse may be had to the very simple measure of applying a compress wet with very cold water to the neck. The compress should be changed as soon as it begins to get warm. The writer has found it very serviceable, and he supposes that it, too, acts on the phrenic nerves. The area of refrigeration is so small that there is no danger of chilling the patient.—*N. Y. Medical Journal.*

